# Supplementary material for: Resonant Inner-Shell Photofragmentation of Adamantane (C10H16)
Source: Molecules. 2023 Jul 19;28(14):5510. doi: 10.3390/molecules28145510 (PMC10384773; doi:10.3390/molecules28145510)
Supplement: Supplementary file 1 [file molecules-28-05510-s001.zip › molecules-2452644-supplementary.pdf]

Resonant inner-shell photofragmentation of Adamantane

Smita Ganguly,[1] Mathieu Gisselbrecht,[1] Per Eng-Johnsson,[1] Raimund Feifel,[2] Sergio Díaz-Tendero,[3,4,5] Eva Muchová,[6] Aleksandar R. Milosavljević,[7] Patrick Rousseau,[8] and Sylvain Maclot[2,9,*]

[1]*Department of Physics, Lund University, 22100 Lund, Sweden*

[2]*Department of Physics, University of Gothenburg, Origovagen 6 B, 41296, Gothenburg, Sweden*

[3]*Department of Chemistry, Universidad Autonoma de Madrid, 28049, Madrid, Spain*

[4]*Institute for Advanced Research in Chemistry (IAdChem), Universidad Autónoma de Madrid, 28049 Madrid, Spain*

[5]*Condensed Matter Physics Center (IFIMAC), Universidad Autónoma de Madrid, 28049 Madrid, Spain*

[6]*Department of Physical Chemistry, University of Chemistry and Technology, Technická 5, 166 28 Prague, Czech Republic*

[7]*Synchrotron SOLEIL, L'Orme de Merisiers, Saint Aubin, BP48, 91192 Gif-sur-Yvette Cedex, France*

[8]*Normandie Univ., ENSICAEN, UNICAEN, CEA, CNRS, CIMAP, 14000, Caen, France*

[9]*Institut Lumiere Matiere UMR 5306, Universite Claude Bernard Lyon 1, CNRS, Univ Lyon, 69100 Villeurbanne, France*

I. SUPPLEMENTARY MATERIAL

A. Theoretical methods

Ground state structure was optimized at the CAM-B3LYP/6-31+g* level. This structure was used for subsequent stick spectra calculations. Calculations were done in Gaussian 09 Revision A02[1]. The nuclear ensemble approach (NEA) was used for modelling of X-ray absorption (XAS). The ensemble of molecular geometries was generated by *ab initio* molecular dynamics (MD, classical description of nuclei and *ab initio* potential energy surface calculated on-the-fly). The *ab initio* potential was calculated at the CAM-B3LYP/6-31+g* level. The total length of MD was 3 ps, the timestep was 0.5 fs, the temperature was set to 300 K using the generalized Langevin equation (GLE) thermostat[2, 3] to account for the nuclear quantum effects. 200 equally distanced frames from molecular dynamics were used for further calculations. In the dynamical simulations, the generated structures lack symmetry, therefore symmetry unrestricted solutions in the *ab initio* calculations were used. The final spectra are a convolution of individual transitions by Gaussian functions with widths significantly smaller than the natural width of the peaks (0.05 eV). The advantage of this approach is that it naturally captures anharmonic and non-Condon effects. However, vibronic progressions are completely neglected[4–6]. Similar approaches have been used from the 1980s and since for electronic spectroscopy in the UV-Vis range or for X-ray absorption[4–11]. The MD simulations were performed in the in-house code ABIN v1.0[12] using the potential calculated in the GPU-accelerated *ab initio* package TeraChem v1.9[13–16].

The XAS spectra were simulated at CVS-EOM-EE-CCSD level with 6-31g* and 6-31+g basis sets. The employed basis sets are rather limited, however, the adamantane molecule contains 10 heavy atoms which makes the use of extended basis sets of aug-cc-pVXZ quality computationally unfeasible. The spectra were modelled also at the TDDFT level using restricted orbital space[17, 18] at the CAM-B3LYP level using 6-31g*, 6-31+g*, 6-31++g** and cc-pCVTZ basis sets and at BMK level with 6-311++g** basis set. The cc-pCVTZ basis set is appropriate for the core excited states and provides an accurate benchmark[19, 20].

The valence band were modelled within the EOM-IP-CCSD approach with the 6-31g*

* sylvain.maclot@univ-lyon1.fr

and 6-31+g* basis sets which provide reasonable agreement with vertical valence ionization energies. For the resonant Auger spectra modelling, the classical two-step model was employed. The excited states were modelled at the CVS-EOM-EE-CCSD/6-31g* level and the final states were described at the EOM-IP-CCSD with the same basis set, e.g. the Feschbach-Fano approach introduced by W. Skomorowski and A. I. Krylov[21, 22] was employed. The outgoing electron was simulated as a plane wave. Since the lowest energy part of the measured resonant Auger spectra corresponds to the participator Auger decay, the Feschbach-Fano approach provides an ideal tool. The used basis set is modest; however, the main focus of the paper is on the description of changes between the two types of carbon core excitations, therefore we can assume that the absolute error is the same for both transitions and the relative differences should be correctly captured. The spectra calculations were performed in Q-Chem 6.0[23].

Understanding of the resonant Auger spectra of a molecule requires a proper description of both the core and valence electronic structure because both is involved in the processes. While the core excitation is an initial step of the resonant Auger process, the valence band electronic structure is reflected in the participator decay process.

XAS spectrum. The XAS modelling is of key importance for interpretation of the resonant Auger spectra because it provides an invaluable insight into the character and energies of the transitions that are initially excited in the resonant experiment. The spectra were simulated at the CVS-EOM-EE-CCSD level with 6-31g* and 6-31+g* basis sets and at the TDDFT/CAM-B3LYP level using the 6-31++g**, 6-31+g* and 6-31g* basis sets and at the BMK/6-311++g** level. The spectra shifted to match the experimentally observed energy of the first peak maximum at 287.1 eV are shown in the panel (a) of the Figure S1.

Since the calculations were performed with a modest basis sets, the error can be as large as $\approx$2eV for the CVS-EOM-EE-CCSD calculations. TDDFT has generally much larger error, yet this is anticipated; the relative state energies are, however, generally well represented. As can be inferred from the Figure S1, the spectral shape and spacing between the first and second peak is reasonably reproduced even if the modest basis sets are used. The lower energy peak corresponds to a combination of excitations from C 1s to valence CH and CH_2 orbitals, the intensity of the C 1s $\rightarrow$ valence CH orbital transition is higher. The character of the transitions is depicted in Figure S2 in terms of natural transition orbitals (NTO) calculated at the CVS-EOM-EE-CCSD/6-31g* level, the mixed transition corresponds to A

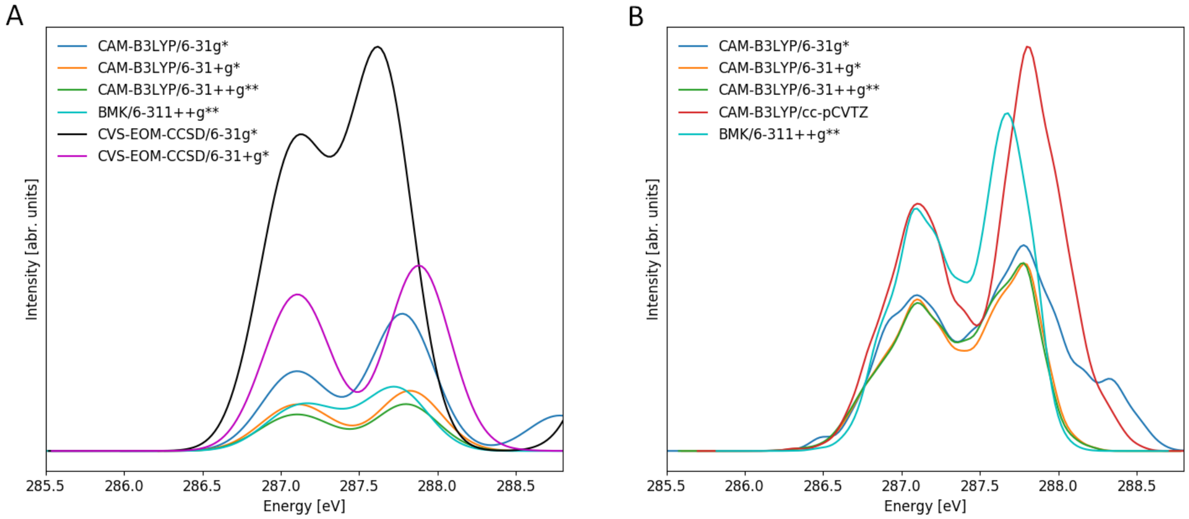

Figure S1. XAS spectra of adamantane. Panel A shows spectra calculated at the TDDFT/CAM-B3LYP, BMK and at CVS-EOM-EE-CCSD levels with various basis sets for the minimum energy structure. The spectra were phenomenologically broadened by 0.2 eV. Panel B shows the spectra within the NEA at various levels of theory calculated for 200 structures from MD. Each point was phenomenologically broadened by 0.05 eV. Spectra were shifted to match the position of the first experimental peak located at 287.1 eV as such: panel (a): CAM-B3LYP/6-31g* → +8.87 eV — CAM-B3LYP/6-31+g* → +10.50 eV — CAM-B3LYP/6-31++g** → +10.67 eV — BMK/6-311++g** → +4.40 eV — CVS-EOM-CCSD/6-31g* → −3.58 eV — CVS-EOM-CCSD/6-31+g* → −1.75 eV and panel (b): CAM-B3LYP/6-31g* → +8.97 eV — CAM-B3LYP/6-31+g* → +10.51 eV — CAM-B3LYP/6-31++g** → +10.98 eV — CAM-B3LYP/cc-pCVTZ → +10.29 eV — BMK/6-311++g** → +4.30 eV

and B. The higher energy peak at 287.6 eV corresponds to the transition C 1s → valence CH orbital and the character of the transition is depicted in Figure S2 as C.

The spectrum simulated within the NEA approach at various levels of theory for 200 geometries is shown in the panel B of Figure S1. As can be seen from the figure, the spectral shape is in agreement with previous work and the energy splitting between the peak maxima also agrees very well. The character of the transitions of the lower energy peak is a combination of CH and CH_2 site transitions, the dominant part is the C 1s → valence CH orbital transition, however, the peak maximum is clearly a convolution of both. The higher energy peak corresponds almost exclusively to the C 1s → valence CH_2 orbital

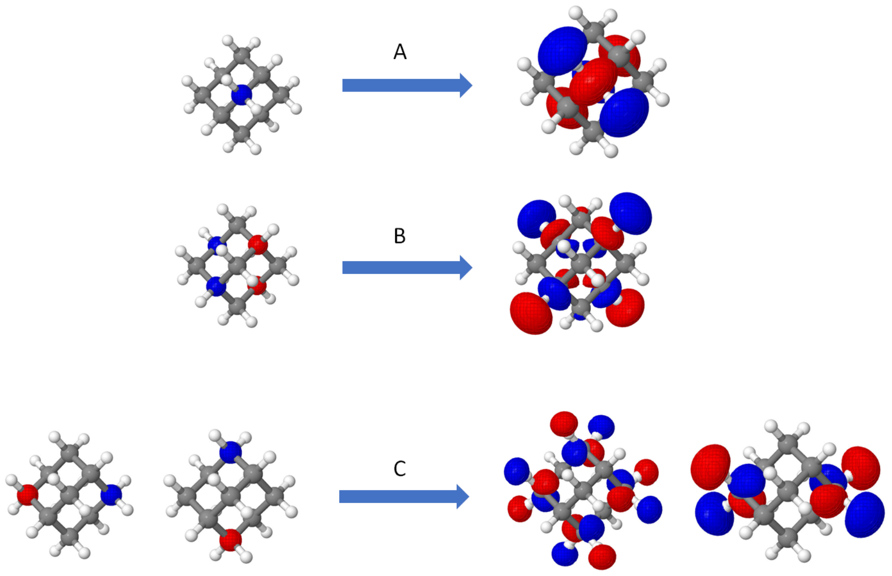

Figure S2. Natural transition orbitals (NTO) calculated at the CVS-EOM-EE-CCSD/6-31g* level for the lowest energy transitions. The lower energy peak in the XAS spectrum corresponds to a combination of excitations from the 1s carbon sites to CH and CH_2 valence orbitals. Excitations are labeled as A and B. The higher energy peak corresponds to the C 1s $\rightarrow$ valence CH_2 orbital excitation, labeled as excitation C.

transitions. The mentioned aspect of a mixed character of the lower energy peak is of importance for resonant Auger spectra simulations.

Valence band XPS. Simulated valence band spectra at the EOM-IP-CCSD level depicted in Figure S3 exhibit a set of peaks between 10-25 eV which are in agreement with the measured experimental spectrum (see the bottom of the Figure 2 in the manuscript). The intensities of the peaks were calculated in terms of Dyson orbital norms. The fact that the spectra are well reproduced even though very modest basis sets were used are crucial for the calculations of the Auger decay rates because we confirm that the description of the final states is reliable.

Resonant Auger spectrum. The resonant Auger spectra can be interpreted with understanding of the character and nature of the transitions contributing to the pre-edge XAS feature. Resonant Auger spectra were recorded with incident photon energies of 287.1 eV and 287.6 eV which according to the calculations and previous literature correspond to the mixed excitation to the CH and partially CH_2 sites (transitions A and B) and to the

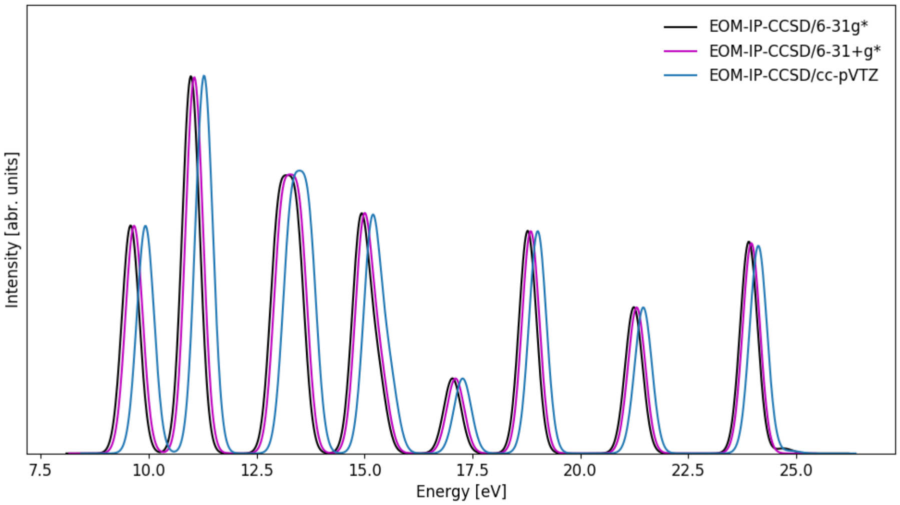

Figure S3. The valence band XPS spectra for the minimum energy structure calculated at the EOM-IP-CCSD level using various basis sets. Each ionization energy was phenomenological Gaussian broadened by 0.2 eV. The intensities were estimated as the norms of the respective Dyson orbitals.

excitation to exclusively CH_2 sites (transition C). The resonant Auger spectra calculated for transitions A, B and C are shown in Figure S4.

Figure S4 shows the spectra in binding energies; the experimental value of 287.1 eV was used for the transitions A and B and the value of 287.6 eV was used for transition C. As can be inferred from the figure, the number of peaks and the spectral shape agree with the IT1 region shown in the experiment in the Figure 1 in the manuscript (the calculations performed within the EOM-CCSD framework describe only the participator Auger decay process (1h)). Generally, the CH site excitation (transition B) exhibit higher decay rates than the CH_2 site excitation (transition C), the only difference is the peak attributed to $2e^{-1}$ which has a lower decay rate than for the CH_2 site excitation. In the experiment, reaching states with t_2 symmetry is higher after CH excitation; this feature is also confirmed in the calculations. There is one more aspect in the interpretation which should be also taken into account – the mixed character of the transitions corresponding to the excitation energy of 287.1 eV. As can be seen in the figure, the decay rates for the excitation A are different from B and C; significantly higher decay rates are observed for $2t_1$,$3e^{-1}$ and $2e^{-1}$. If the decay rates for the A excitation are included in the 287.1 eV excitation, the theoretical resonant

Auger spectrum agrees very well with the experiment, see the Figure 3 in the manuscript.

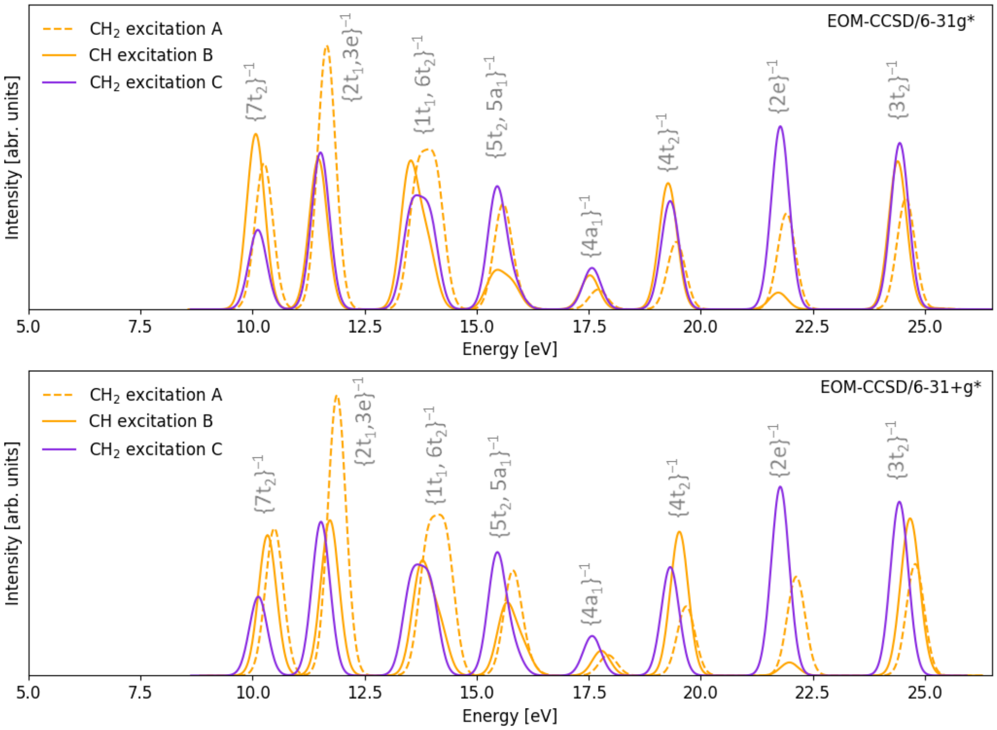

Figure S4. The resonant Auger spectra for the minimum energy structure calculated within the EOM-CCSD framework level using 6-31g* (upper panel) and 6-31+g* (lower panel) basis sets. Each energy was phenomenological Gaussian broadened by 0.2 eV. The spectra in the upper panel were shifted by 4.15 eV and in the lower panel by 2.5 eV to higher energies to match the experimental spectra.

---

[1] M. Frisch, G. Trucks, H. Schlegel, G. Scuseria, M. Robb, J. Cheeseman, G. Scalmani, V. Barone, G. Petersson, H. Nakatsuji, X. Li, M. Caricato, A. Marenich, J. Bloino, B. Janesko, R. Gomperts, B. Mennucci, H. Hratchian, J. Ortiz, A. Izmaylov, J. Sonnenberg, D. Williams-Young, F. Ding, F. Lipparini, F. Egidi, J. Goings, B. Peng, A. Petrone, T. Henderson, D. Ranasinghe, V. Zakrzewski, J. Gao, N. Rega, G. Zheng, W. Liang, M. Hada, M. Ehara, K. Toyota, R. Fukuda, J. Hasegawa, M. Ishida, T. Nakajima, Y. Honda, O. Kitao, H. Nakai,

T. Vreven, K. Throssell, J. J. Montgomery, J. Peralta, F. Ogliaro, M. Bearpark, J. Heyd, E. Brothers, K. Kudin, V. Staroverov, T. Keith, R. Kobayashi, J. Normand, K. Raghavachari, A. Rendell, J. Burant, S. Iyengar, J. Tomasi, M. Cossi, J. Millam, M. Klene, C. Adamo, R. Cammi, J. Ochterski, R. Martin, K. Morokuma, O. Farkas, J. Foresman, and D. Fox, Gaussian 09, revision a.02 (2016).

[2] M. Ceriotti, G. Bussi, and M. Parrinello, Nuclear quantum effects in solids using a colored-noise thermostat, Physical Review Letters **103**, 030603 (2009).

[3] M. Ceriotti, G. Bussi, and M. Parrinello, Colored-noise thermostats àla carte, Journal of Chemical Theory and Computation **6**, 1170 (2010).

[4] R. Crespo-Otero and M. Barbatti, Spectrum simulation and decomposition with nuclear ensemble: Formal derivation and application to benzene, furan and 2-phenylfuran, Theoretical Chemistry Accounts **131**, 1 (2012).

[5] A. Prlj, E. Marsili, L. Hutton, D. Hollas, D. Shchepanovska, D. R. Glowacki, P. Slavíček, and B. F. Curchod, Calculating photoabsorption cross-sections for atmospheric volatile organic compounds, ACS Earth and Space Chemistry **6**, 207 (2022).

[6] F. D. Sala, R. Rousseau, A. Görling, and D. Marx, Quantum and thermal fluctuation effects on the photoabsorption spectra of clusters, Physical Review Letters **92**, 183401 (2004).

[7] S. Y. Lee, R. C. Brown, and E. J. Heller, Multidimensional reflection approximation: Application to the photodissociation of polyatomics, Journal of Physical Chemistry **87**, 2045 (1983).

[8] S. Y. Lee, Semiclassical theory of radiation interacting with a molecule, The Journal of Chemical Physics **76**, 3064 (1998).

[9] P. C. D. Couto, D. Hollas, and P. Slavíček, On the performance of optimally tuned range-separated hybrid functionals for x-ray absorption modeling, Journal of Chemical Theory and Computation **11**, 3234 (2015).

[10] W. Zeng, S. Gong, C. Zhong, and C. Yang, Prediction of oscillator strength and transition dipole moments with the nuclear ensemble approach for thermally activated delayed fluorescence emitters, Journal of Physical Chemistry C **123**, 10081 (2019).

[11] M. Ončák, L. Šištík, and P. Slavíček, Can theory quantitatively model stratospheric photolysis? ab initio estimate of absolute absorption cross sections of cloocl, The Journal of Chemical Physics **133**, 174303 (2010).

[12] P. Slavíček, M. Ončák, D. Hollas, and O. Svoboda, Abin, version 1.0, https://github.com/PHOTOX/ABIN.

[13] S. Seritan, C. Bannwarth, B. S. Fales, E. G. Hohenstein, C. M. Isborn, S. I. Kokkila-Schumacher, X. Li, F. Liu, N. Luehr, J. W. Snyder, C. Song, A. V. Titov, I. S. Ufimtsev, L. P. Wang, and T. J. Martínez, Terachem: A graphical processing unit-accelerated electronic structure package for large-scale ab initio molecular dynamics, Wiley Interdisciplinary Reviews: Computational Molecular Science **11**, e1494 (2021).

[14] I. S. Ufimtsev and T. J. Martínez, Quantum chemistry on graphical processing units. 1. strategies for two-electron integral evaluation, Journal of Chemical Theory and Computation **4**, 222 (2008).

[15] I. S. Ufimtsev and T. J. Martinez, Quantum chemistry on graphical processing units. 3. analytical energy gradients, geometry optimization, and first principles molecular dynamics, Journal of Chemical Theory and Computation **5**, 2619 (2009).

[16] I. S. Ufimtsev and T. J. Martinez, Quantum chemistry on graphical processing units. 2. direct self-consistent-field implementation, Journal of Chemical Theory and Computation **5**, 1004 (2009).

[17] S. DeBeer George, T. Petrenko, and F. Neese, Time-dependent density functional calculations of ligand K-edge X-ray absorption spectra, Inorganica Chim. Acta **361**, 965 (2008).

[18] M. Stener, G. Fronzoni, and M. de Simone, Time dependent density functional theory of core electrons excitations, Chem. Phys. Lett. **373**, 115 (2003).

[19] A. I. Krylov, Equation-of-motion coupled-cluster methods for open-shell and electronically excited species: The hitchhiker's guide to fock space, Annual Review of Physical Chemistry **59**, 433 (2008).

[20] T. Fransson, I. E. Brumboiu, M. L. Vidal, P. Norman, S. Coriani, and A. Dreuw, Xaboom: An x-ray absorption benchmark of organic molecules based on carbon, nitrogen, and oxygen 1s $\rightarrow$ * transitions, Journal of Chemical Theory and Computation **17**, 1618 (2021), pMID: 33544612, https://doi.org/10.1021/acs.jctc.0c01082.

[21] W. Skomorowski and A. I. Krylov, Feshbach-fano approach for calculation of auger decay rates using equation-of-motion coupled-cluster wave functions. i. theory and implementation, The Journal of Chemical Physics **154**, 084124 (2021).

[22] W. Skomorowski and A. I. Krylov, Feshbach–fano approach for calculation of auger decay

rates using equation-of-motion coupled-cluster wave functions. ii. numerical examples and benchmarks, The Journal of Chemical Physics **154**, 084125 (2021).

[23] E. Epifanovsky, A. T. Gilbert, X. Feng, J. Lee, Y. Mao, N. Mardirossian, P. Pokhilko, A. F. White, M. P. Coons, A. L. Dempwolff, Z. Gan, D. Hait, P. R. Horn, L. D. Jacobson, I. Kaliman, J. Kussmann, A. W. Lange, K. U. Lao, D. S. Levine, J. Liu, S. C. McKenzie, A. F. Morrison, K. D. Nanda, F. Plasser, D. R. Rehn, M. L. Vidal, Z. Q. You, Y. Zhu, B. Alam, B. J. Albrecht, A. Aldossary, E. Alguire, J. H. Andersen, V. Athavale, D. Barton, K. Begam, A. Behn, N. Bellonzi, Y. A. Bernard, E. J. Berquist, H. G. Burton, A. Carreras, K. Carter-Fenk, R. Chakraborty, A. D. Chien, K. D. Closser, V. Cofer-Shabica, S. Dasgupta, M. D. Wergifosse, J. Deng, M. Diedenhofen, H. Do, S. Ehlert, P. T. Fang, S. Fatehi, Q. Feng, T. Friedhoff, J. Gayvert, Q. Ge, G. Gidofalvi, M. Goldey, J. Gomes, C. E. González-Espinoza, S. Gulania, A. O. Gunina, M. W. Hanson-Heine, P. H. Harbach, A. Hauser, M. F. Herbst, M. H. Vera, M. Hodecker, Z. C. Holden, S. Houck, X. Huang, K. Hui, B. C. Huynh, M. Ivanov, Ádám Jász, H. Ji, H. Jiang, B. Kaduk, S. Kähler, K. Khistyaev, J. Kim, G. Kis, P. Klunzinger, Z. Koczor-Benda, J. H. Koh, D. Kosenkov, L. Koulias, T. Kowalczyk, C. M. Krauter, K. Kue, A. Kunitsa, T. Kus, I. Ladjánszki, A. Landau, K. V. Lawler, D. Lefrancois, S. Lehtola, R. R. Li, Y. P. Li, J. Liang, M. Liebenthal, H. H. Lin, Y. S. Lin, F. Liu, K. Y. Liu, M. Loipersberger, A. Luenser, A. Manjanath, P. Manohar, E. Mansoor, S. F. Manzer, S. P. Mao, A. V. Marenich, T. Markovich, S. Mason, S. A. Maurer, P. F. McLaughlin, M. F. Menger, J. M. Mewes, S. A. Mewes, P. Morgante, J. W. Mullinax, K. J. Oosterbaan, G. Paran, A. C. Paul, S. K. Paul, F. Pavošević, Z. Pei, S. Prager, E. I. Proynov, Ádám Rák, E. Ramos-Cordoba, B. Rana, A. E. Rask, A. Rettig, R. M. Richard, F. Rob, E. Rossomme, T. Scheele, M. Scheurer, M. Schneider, N. Sergueev, S. M. Sharada, W. Skomorowski, D. W. Small, C. J. Stein, Y. C. Su, E. J. Sundstrom, Z. Tao, J. Thirman, G. J. Tornai, T. Tsuchimochi, N. M. Tubman, S. P. Veccham, O. Vydrov, J. Wenzel, J. Witte, A. Yamada, K. Yao, S. Yeganeh, S. R. Yost, A. Zech, I. Y. Zhang, X. Zhang, Y. Zhang, D. Zuev, A. Aspuru-Guzik, A. T. Bell, N. A. Besley, K. B. Bravaya, B. R. Brooks, D. Casanova, J. D. Chai, S. Coriani, C. J. Cramer, G. Cserey, A. E. Deprince, R. A. Distasio, A. Dreuw, B. D. Dunietz, T. R. Furlani, W. A. Goddard, S. Hammes-Schiffer, T. Head-Gordon, W. J. Hehre, C. P. Hsu, T. C. Jagau, Y. Jung, A. Klamt, J. Kong, D. S. Lambrecht, W. Liang, N. J. Mayhall, C. W. McCurdy, J. B. Neaton, C. Ochsenfeld, J. A. Parkhill, R. Peverati, V. A. Rassolov, Y. Shao, L. V. Slipchenko, T. Stauch, R. P. Steele, J. E.

Subotnik, A. J. Thom, A. Tkatchenko, D. G. Truhlar, T. V. Voorhis, T. A. Wesolowski, K. B. Whaley, H. L. Woodcock, P. M. Zimmerman, S. Faraji, P. M. Gill, M. Head-Gordon, J. M. Herbert, and A. I. Krylov, Software for the frontiers of quantum chemistry: An overview of developments in the q-chem 5 package, The Journal of Chemical Physics **155**, 084801 (2021).
